# Supplementary material for: Differences in Mechanical, Electrical and Calcium Transient Performance of the Isolated Right Atrial and Ventricular Myocardium of Guinea Pigs at Different Preloads (Lengths)
Source: Int J Mol Sci. 2023 Oct 24;24(21):15524. doi: 10.3390/ijms242115524 (PMC10650485; doi:10.3390/ijms242115524)
Supplement: Supplementary file 1 [file ijms-24-15524-s001.zip › ijms-2665073-supplementary.pdf]

Table S1. TTP RV (**Figure 4A**)

ANOVA Friedman results				
**factor**	**Kendall's coefficient of concordance**	**degrees of freedom**	χ^2	***p*-value**
Length, L/L_{max}	0.8215	4	36.1461	2.7E-07

p-values - results of Siegel and Castellan´s all-pairs comparisons post-hoc test with closed method based on Simes tests (non-negative) to adjust *p*-values

Length, L/L_{max}	**0.8**	**0.85**	**0.9**	**0.95**	**1.0**
**0.8**	1	1.5E-1	3.8E-3	3.1E-5	2.1E-6
**0.85**	1.5E-1	1	1.5E-1	7.5E-3	1.2E-3
**0.9**	3.8E-3	1.5E-1	1	2.0E-1	7.2E-2
**0.95**	3.1E-5	7.5E-3	2.0E-1	1	5.0E-1
**1.0**	2.1E-6	1.2E-3	7.2E-2	5.0E-1	1

$p < 0.05$ are highlighted in red.

Table S2. TTP RA (**Figure 4A**)

ANOVA Friedman results				
**factor**	**Kendall's coefficient of concordance**	**degrees of freedom**	χ^2	***p*-value**
Length, L/L_{max}	0.9003	4	21.6068	2.4E-04

p-values - results of Siegel and Castellan´s all-pairs comparisons post-hoc test with closed method based on Simes tests (non-negative) to adjust *p*-values

Length, L/L_{max}	**0.8**	**0.85**	**0.9**	**0.95**	**1.0**
**0.8**	1	4.0E-01	2.6E-02	8.6E-03	5.9E-04
**0.85**	4.0E-01	1	1.7E-01	7.2E-02	8.6E-03
**0.9**	2.6E-02	1.7E-01	1	6.5E-01	2.1E-01
**0.95**	8.6E-03	7.2E-02	6.5E-01	1	3.9E-01
**1.0**	5.9E-04	8.6E-03	2.1E-01	3.9E-01	1

$p < 0.05$ are highlighted in red.

Table S3. T50 RV (**Figure 4B**)

ANOVA Friedman results				
**factor**	**Kendall's coefficient of concordance**	**degrees of freedom**	χ^2	***p*-value**
Length, L/L_{max}	0.6398	4	28.1517	1.2E-05

p-values - results of Siegel and Castellan´s all-pairs comparisons post-hoc test with closed method based on Simes tests (non-negative) to adjust *p*-values

Length, L/L_{max}	**0.8**	**0.85**	**0.9**	**0.95**	**1.0**
**0.8**	1	2.5E-01	5.2E-02	6.4E-03	1.2E-05
**0.85**	2.5E-01	1	3.8E-01	1.1E-01	1.8E-03
**0.9**	5.2E-02	3.8E-01	1	3.8E-01	2.1E-02
**0.95**	6.4E-03	1.1E-01	3.8E-01	1.	1.1E-01
**1.0**	1.2E-05	1.8E-03	2.1E-02	1.1E-01	1

$p < 0.05$ are highlighted in red.

Table S4. T50 RA (**Figure 4B**)

ANOVA Friedman results

factor	Kendall's coefficient of concordance	degrees of freedom	χ^2	*p*-value
Length, L/L_{max}	1	4	24	8.0E-05

p-values - results of Siegel and Castellan´s all-pairs comparisons post-hoc test with closed method based on Simes tests (non-negative) to adjust *p*-values

Length, L/L_{max}	**0.8**	**0.85**	**0.9**	**0.95**	**1.0**
**0.8**	1	2.7E-01	4.7E-02	3.4E-03	1.2E-04
**0.85**	2.7E-01	1	2.7E-01	4.7E-02	3.4E-03
**0.9**	4.7E-02	2.7E-01	1	2.7E-01	4.7E-02
**0.95**	3.4E-03	4.7E-02	2.7E-01	1	2.7E-01
**1.0**	1.2E-04	3.4E-03	4.7E-02	2.7E-01	1

$p < 0.05$ are highlighted in red.

Table S5. Normalised values of maximal rates of tension development RV (**Figure 4C**)

ANOVA Friedman results

factor	Kendall's coefficient of concordance	degrees of freedom	χ^2	*p*-value
Length, L/L_{max}	0.7174	4	31.5636	2.3E-06

p-values - results of Siegel and Castellan´s all-pairs comparisons post-hoc test with closed method based on Simes tests (non-negative) to adjust *p*-values

Length, L/L_{max}	**0.8**	**0.85**	**0.9**	**0.95**	**1.0**
**0.8**	1	3.1E-01	6.0E-03	2.6E-04	2.4E-05
**0.85**	3.1E-01	1	9.8E-02	6.0E-03	9.1E-04
**0.9**	6.0E-03	9.8E-02	1	3.1E-01	1.1E-01
**0.95**	2.6E-04	6.0E-03	3.1E-01	1	5.0E-01
**1.0**	2.4E-05	9.1E-04	1.1E-01	5.0E-01	1

$p < 0.05$ are highlighted in red.

Table S6. Normalised values of maximal rates of tension development RA (**Figure 4C**)

ANOVA Friedman results

factor	Kendall's coefficient of concordance	degrees of freedom	χ^2	*p*-value
Length, L/L_{max}	0.9722	4	23.3333	1.1E-04

p-values - results of Siegel and Castellan´s all-pairs comparisons post-hoc test with closed method based on Simes tests (non-negative) to adjust *p*-values

Length, L/L_{max}	**0.8**	**0.85**	**0.9**	**0.95**	**1.0**
**0.8**	1	3.0E-01	4.4E-02	6.4E-03	1.2E-04
**0.85**	3.0E-01	1	2.5E-01	7.4E-02	5.1E-03
**0.9**	4.4E-02	2.5E-01	1	4.7E-01	7.4E-02
**0.95**	6.4E-03	7.4E-02	4.7E-01	1	2.5E-01
**1.0**	1.2E-04	5.1E-03	7.4E-02	2.5E-01	1

$p < 0.05$ are highlighted in red.

Table S7. Normalised values of maximal rates of tension relaxation RV (**Figure 4D**)

ANOVA Friedman results				
**factor**	**Kendall's coefficient of concordance**	**degrees of freedom**	χ^2	***p*-value**
Length, L/L_{max}	0.8017	4	35.2727	4.1E-07

p-values - results of Siegel and Castellan´s all-pairs comparisons post-hoc test with closed method based on Simes tests (non-negative) to adjust *p*-values

Length, L/L_{max}	**0.8**	**0.85**	**0.9**	**0.95**	**1.0**
**0.8**	1	3.8E-01	1.0E-01	1.1E-03	1.5E-06
**0.85**	3.8E-01	1	4.2E-01	2.1E-02	8.0E-05
**0.9**	1.0E-01	4.2E-01	1	1.0E-01	1.1E-03
**0.95**	1.1E-03	2.1E-02	1.0E-01	1	1.0E-01
**1.0**	1.5E-06	8.0E-05	1.1E-03	1.0E-01	1

$p < 0.05$ are highlighted in red.

Table S8. Normalised values of maximal rates of tension relaxation RA (**Figure 4D**)

ANOVA Friedman results				
**factor**	**Kendall's coefficient of concordance**	**degrees of freedom**	χ^2	***p*-value**
Length, L/L_{max}	1	4	24	8.0E-05

p-values - results of Siegel and Castellan´s all-pairs comparisons post-hoc test with closed method based on Simes tests (non-negative) to adjust *p*-values

**0.8**	1	2.7E-01	4.7E-02	3.4E-03	1.2E-04
**0.85**	2.7E-01	1	2.7E-01	4.7E-02	3.4E-03
**0.9**	4.7E-02	2.7E-01	1	2.7E-01	4.7E-02
**0.95**	3.4E-03	4.7E-02	2.7E-01	1	2.7E-01
**1.0**	1.2E-04	3.4E-03	4.7E-02	2.7E-01	1
**0.8**	1	2.7E-01	4.7E-02	3.4E-03	1.2E-04

$p < 0.05$ are highlighted in red.

Table S9. APD90 RV (**Figure 5B**)

ANOVA Friedman results				
**factor**	**Kendall's coefficient of concordance**	**degrees of freedom**	χ^2	***p*-value**
Length, L/L_{max}	0.0477	4	2.4839	0.65

Table S10. APD90 RA (**Figure 5B**)

ANOVA Friedman results				
**factor**	**Kendall's coefficient of concordance**	**degrees of freedom**	χ^2	***p*-value**
Length, L/L_{max}	0.1139	4	5.4679	0.24

Table S11. T70Ca RV (**Figure 6B**)

ANOVA Friedman results

factor	Kendall's coefficient of concordance	degrees of freedom	χ^2	*p*-value
Length, L/L_{max}	0.2564	4	11.2811	2.4E-02

p-values - results of Siegel and Castellan´s all-pairs comparisons post-hoc test with closed method based on Simes tests (non-negative) to adjust *p*-values

Length, L/L_{max}	**0.8**	**0.85**	**0.9**	**0.95**	**1.0**
**0.8**	1	8.9E-01	8.6E-02	8.6E-02	8.6E-02
**0.85**	8.9E-01	1	9.8E-02	8.6E-02	8.6E-02
**0.9**	8.6E-02	9.8E-02	1	8.9E-01	8.9E-01
**0.95**	8.6E-02	8.6E-02	8.9E-01	1	8.9E-01
**1.0**	8.6E-02	8.6E-02	8.9E-01	8.9E-01	1

$p < 0.05$ are highlighted in red.

Table S12. T70Ca RA (**Figure 6B**)

ANOVA Friedman results

factor	Kendall's coefficient of concordance	degrees of freedom	χ^2	*p*-value
Length, L/L_{max}	0.6918	6	24.9036	3.6E-04

p-values - results of Siegel and Castellan´s all-pairs comparisons post-hoc test with closed method based on Simes tests (non-negative) to adjust *p*-values

Length, L/L_{max}	**0.7**	**0.75**	**0.8**	**0.85**	**0.9**	**0.95**	**1.0**
**0.7**	1	7.8E-01	3.7E-01	1.9E-01	1.7E-02	2.2E-02	9.4E-03
**0.75**	7.8E-01	1	2.6E-01	1.4E-01	9.4E-03	1.1E-02	6.5E-03
**0.8**	3.7E-01	2.6E-01	1	6.9E-01	1.7E-01	1.8E-01	8.2E-02
**0.85**	1.9E-01	1.4E-01	6.9E-01	1	3.1E-01	3.6E-01	1.8E-01
**0.9**	1.7E-02	9.4E-03	1.7E-01	3.1E-01	1	8.9E-01	7.6E-01
**0.95**	2.2E-02	1.1E-02	1.8E-01	3.6E-01	8.9E-01	1	6.9E-01
**1.0**	9.4E-03	6.5E-03	8.2E-02	1.8E-01	7.6E-01	6.9E-01	1

$p < 0.05$ are highlighted in red.

Table S13. Amplitude of the difference curve in the III phase of the “CaT difference curve” RV (**Figure 7C**)

ANOVA Friedman results

factor	Kendall's coefficient of concordance	degrees of freedom	χ^2	*p*-value
Length, L/L_{max}	0.32	3	9.6	2.2E-02

p-values - results of Siegel and Castellan´s all-pairs comparisons post-hoc test with closed method based on Simes tests (non-negative) to adjust *p*-values

Length, L/L_{max}	**0.85**	**0.9**	**0.95**	**1.0**
**0.85**	1	5.9E-01	8.4E-02	2.6E-02
**0.9**	5.9E-01	1	2.1E-01	6.2E-02
**0.95**	8.4E-02	2.1E-01	1	4.9E-01
**1.0**	2.6E-02	6.2E-02	4.9E-01	1

$p < 0.05$ are highlighted in red.

Table S14. Amplitude of the difference curve in the III phase of the "CaT difference curve" RA (**Figure 7C**)

ANOVA Friedman results

factor	Kendall's coefficient of concordance	degrees of freedom	χ^2	*p*-value
Length, L/L_{max}	0.9444	3	17	7.07E-04

p-values - results of Siegel and Castellan´s all-pairs comparisons post-hoc test with closed method based on Simes tests (non-negative) to adjust *p*-values

Length, L/L_{max}	**0.85**	**0.9**	**0.95**	**1.0**
**0.85**	1	3.7E-01	2.8E-02	8.6E-04
**0.9**	3.7E-01	1	1.8E-01	1.1E-02
**0.95**	2.8E-02	1.8E-01	1	2.2E-01
**1.0**	8.6E-04	1.1E-02	2.2E-01	1

$p < 0.05$ are highlighted in red.

Table S15. Relative area (in %) calculated under the difference curve in the III phase of the "CaT difference curve" RV (**Figure 7D**)

ANOVA Friedman results

factor	Kendall's coefficient of concordance	degrees of freedom	χ^2	*p*-value
Length, L/L_{max}	0.416	3	12.48	5.9E-03

p-values - results of Siegel and Castellan´s all-pairs comparisons post-hoc test with closed method based on Simes tests (non-negative) to adjust *p*-values

Length, L/L_{max}	**0.85**	**0.9**	**0.95**	**1.0**
**0.85**	1	2.6E-01	7.8E-02	2.3E-03
**0.9**	2.6E-01	1	4.1E-01	6.2E-02
**0.95**	7.8E-02	4.1E-01	1	2.1E-01
**1.0**	2.3E-03	6.2E-02	2.1E-01	1

$p < 0.05$ are highlighted in red.

Table S16. Relative area (in %) calculated under the difference curve in the III phase of the "CaT difference curve" RA (**Figure 7D**)

ANOVA Friedman results

factor	Kendall's coefficient of concordance	degrees of freedom	χ^2	*p*-value
Length, L/L_{max}	0.8778	3	15.8	1.2E-03

p-values - results of Siegel and Castellan´s all-pairs comparisons post-hoc test with closed method based on Simes tests (non-negative) to adjust *p*-values

Length, L/L_{max}	**0.85**	**0.9**	**0.95**	**1.0**
**0.85**	1	2.6E-01	5.1E-02	8.6E-04
**0.9**	2.6E-01	1	2.6E-01	2.2E-02
**0.95**	5.1E-02	2.6E-01	1	1.8E-01
**1.0**	8.6E-04	2.2E-02	1.8E-01	1

$p < 0.05$ are highlighted in red.
